# Development and evaluation of a risk prediction model for enteral nutrition feeding intolerance in intensive care units

**DOI:** 10.3389/fnut.2025.1667046

**Published:** 2025-09-18

**Authors:** Xiaohua Cao, Hua Wang, Yinling Song, Xiangru Yan, Wenjuan Wu, Wenqiang Li, Lulu Chen

**Affiliations:** ^1^Department of Intensive Care Medicine, Jining No. 1 People’s Hospital, Jining, China; ^2^Department of Hematology, Jining No. 1 People’s Hospital, Jining, China

**Keywords:** enteral nutrition feeding intolerance, ENFI, intensive care units, ICUs, critically ill patients, risk prediction model

## Abstract

**Background:**

Patients in intensive care units (ICUs) who receive enteral nutrition (EN) treatment frequently experience feeding intolerance (FI), which, if not promptly managed, can adversely affect treatment outcomes and overall prognosis. This study aims to identify the risk factors associated with enteral nutrition feeding intolerance (ENFI) in critically ill ICU patients and to develop a predictive model to assess the risk of ENFI.

**Methods:**

This study enrolled 144 patients, who were categorized into an ENFI group and a non-ENFI group. Variable selection for model development was conducted through univariate analysis, multicollinearity testing, and binary logistic regression. Based on the logistic regression results, a visual predictive model for ENFI risk was constructed using a nomogram. The model’s discriminative performance was evaluated using the area under the curve (AUC), sensitivity, specificity, positive predictive value (PPV), and negative predictive value (NPV). Internal validation was performed using the bootstrap method with 1,000 resamples of the original dataset. A calibration curve was generated, and the Hosmer–Lemeshow goodness-of-fit test was applied to assess the model’s calibration accuracy.

**Results:**

Based on the results of the binary logistic regression analysis, a nomogram model was developed to predict enteral nutrition feeding intolerance (ENFI) in critically ill ICU patients. The model incorporated five variables: Acute Physiology and Chronic Health Evaluation II (APACHE II) score, mechanical ventilation (MV), albumin (ALB), intra-abdominal pressure (IAP), and EN start time. AUC was 0.800 (95% confidence interval, 0.725–0.875), with a cutoff value of 0.306. The model demonstrated a sensitivity of 82.5%, specificity of 72.4%, positive predictive value (PPV) of 67.2%, and negative predictive value (NPV) of 86.3%. Following internal validation using the bootstrap method, the Hosmer–Lemeshow goodness-of-fit test produced a χ^2^ value of 2.9954 (*p* = 0.9346). The lack of statistically significant deviation between the predicted and observed risk values indicates that the model demonstrates good calibration and accurately reflects the actual risk of ENFI.

**Conclusion:**

The model demonstrated good predictive performance and can effectively assess the risk of ENFI in critically ill ICU patients.

## 1 Introduction

Patients admitted to intensive care units (ICUs) are at high risk of malnutrition due to pathophysiological changes such as increased inflammation, heightened catabolism, decreased nutrient absorption, and reduced intake of macronutrients, which may occur in conditions like severe trauma, shock, and systemic infections (1). In the absence of timely and adequate nutritional treatment, malnutrition can significantly worsen clinical outcomes and increase mortality (2). Early enteral nutrition (EN) treatment is now recognized as a cornerstone of nutritional therapy in critically ill patients. When clinically feasible, EN is preferred over parenteral nutrition (PN) because it helps maintain gastrointestinal integrity and function, while also supporting metabolic and nutritional requirements (3). According to clinical guidelines issued by the Society of Critical Care Medicine (SCCM) and the American Society for Parenteral and Enteral Nutrition (ASPEN), EN should be initiated within 24–48 h of ICU admission for patients who are unable to achieve adequate oral intake, provided there are no contraindications (4).

Despite its benefits, the administration of EN is not without complications. One of the most common is enteral nutrition feeding intolerance (ENFI), which refers to a range of gastrointestinal symptoms—including abdominal distension, vomiting, diarrhea, and constipation—that may lead to the reduction, interruption, or cessation of EN. As a result, patients may fail to reach the target caloric intake of 20 kcal/kg/day within the first 72 h of EN initiation (5). The reported incidence of ENFI in ICU patients receiving EN ranges from 41.27 to 73.60% (6).

ENFI can result in inadequate nutrient delivery, leading to worsened nutritional status, increased risk of infections and complications, prolonged ICU stays, and even increased mortality. Several studies have demonstrated an association between ENFI and adverse outcomes. For example, Gungabissoon et al. (7) found that ENFI is linked to higher mortality, while Hu et al. (8) showed that ICU patients with persistent ENFI had significantly lower 28-day survival rates. Furthermore, ENFI occurring within the first week of ICU admission was identified as an independent risk factor for both 28-day and 90-day mortality.

Given the high prevalence and clinical implications of ENFI, there is a pressing need for tools to identify patients at risk for developing feeding intolerance early in the course of critical illness. Therefore, this study aims to explore the key risk factors associated with ENFI in ICU patients, identify reasonable indicators for the initiation of EN, and construct a predictive model for assessing the risk of ENFI. The resulting model is intended to assist healthcare professionals in the early identification, prevention, and targeted management of ENFI, ultimately improving the safety and effectiveness of EN therapy in the ICU setting.

## 2 Methods

### 2.1 Study participants

This study enrolled critically ill patients admitted to the Intensive Care Unit (ICU) of Jining First People’s Hospital between November 2024 and February 2025, based on the following eligibility criteria.

#### 2.1.1 Inclusion criteria

1. ICU patients identified as being at nutritional risk and meeting the clinical indications for enteral nutrition (EN) therapy.
2. Patients assessed to be at nutritional risk according to at least one of the following tools.

- NRS-2002 score ≥ 3 (9).
- Original NUTRIC score ≥ 6 (10).
- Modified NUTRIC (mNUTRIC) score ≥ 5 (11).

1. Provision of informed consent by the patient or their legally authorized representative, with voluntary agreement to participate in the study.

#### 2.1.2 Exclusion criteria

1. Patients who were not at nutritional risk.
2. Patients with severe clinical instability precluding the initiation of EN.
3. Patients who had undergone gastrointestinal surgery or were diagnosed with active intestinal infections.

Prior to the initiation of the study, ethical approval (JNRM-2025-KY-013) was obtained from the Institutional Review Board of Jining No. 1 People’s Hospital.

#### 2.1.3 EN administration protocol and monitoring

EN was administered continuously over 24 h via a nasogastric or nasojejunal feeding tube in accordance with the ICU protocol, to minimize the risk of gastrointestinal complications. The infusion volume was gradually increased according to individual tolerance and nutritional requirements. Prior to initiating nutritional support, routine monitoring of liver and kidney function, as well as metabolic indicators such as blood glucose, blood lipids, and electrolytes, was performed. Energy requirements were estimated at 25–30 kcal·kg^−1^·d^−1^, and protein intake for critically ill patients was set at 1.2–2.0 g·kg^−1^·d^−1^ (12). According to the latest ASPEN guidelines, patients with severe trauma or critical illness were provided with approximately 70% of the target energy during the first week. Nutritional therapy was initiated with moderate feeding (50–70% of the target), with the goal of achieving at least 80% of the estimated energy and protein requirements within 48–72 h (13). Underfeeding was defined as <80% of the prescribed target, while overfeeding was defined as >120% (14). To monitor tolerance and ensure safe nutritional delivery, blood glucose levels were measured at the start of ICU admission or medical nutrition treatment, and subsequently at least once every 4 h within the first 2 days. Gastric residual volume (GRV) was assessed at least every 6 h to evaluate gastric emptying and detect feeding intolerance. These measures were implemented to optimize patient safety and nutritional adequacy.

The diagnostic criteria for ENFI were adopted from the guidelines proposed by McClave et al. (15). A diagnosis of ENFI was made if one or more of the following criteria were met:

1. Gastrointestinal adverse symptoms, including vomiting or regurgitation, abdominal distension, diarrhea, gastrointestinal bleeding, diminished or absent bowel sounds, constipation, a gastric residual volume ≥500 mL within 24 h, or any other clinical sign indicating intolerance to enteral nutrition.
2. Failure to achieve the target energy intake of 83.68 kJ/kg/day (equivalent to 20 kcal/kg/day) within 72 h of initiating EN.
3. Discontinuation of EN due to any clinical reason.

Patients meeting at least one of the above criteria were considered to have developed ENFI.

### 2.2 Sample size estimation and variable selection

#### 2.2.1 Sample size estimation

The required sample size for this study was estimated using the events per variable (EPV) method, a standard approach in binary logistic regression analysis. An EPV of 10 was used, with an anticipated inclusion of 4 to 6 predictor variables in the final model. According to previously published studies, the incidence of ENFI in critically ill patients ranges from 41.27 to 73.60%. Allowing for a 10% potential sample loss, the minimum and maximum required sample sizes were calculated as follows:

- Minimum: $\frac{10\times 4}{0.736}$ × (1 + 10%) ≈ 60
- Maximum: $\frac{10\times 6}{0.4127}$ × (1 + 10%) ≈ 160

Ultimately, 144 patients were included in the study, which falls within the estimated range and meets the minimum requirement for model development.

#### 2.2.2 Variable selection

##### 2.2.2.1 General information

Includes age, gender, and body mass index (BMI). BMI was calculated as weight in kilograms divided by height in meters squared (kg/m^2^).

##### 2.2.2.2 Clinical data

Clinical variables included the Acute Physiology and Chronic Health Evaluation II (APACHE II) score, presence of chronic comorbidities, use of mechanical ventilation (MV), administration of antibiotics, sedatives, analgesics, and potassium supplements, and serum albumin (ALB) levels.

##### 2.2.2.3 Nutrition-related data and laboratory indicators

Includes nutritional risk score (NRS2002), aspiration risk score, intra-abdominal pressure (IAP), EN start time, EN feeding method, etc.

### 2.3 Data analysis

#### 2.3.1 Statistical methods

Patients with missing values for any of the key predictor or outcome variables were excluded from the analysis. All statistical analyses were performed using SPSS version 26.0 (IBM Corp., Armonk, NY, United States) and R version 4.3.1 (R Foundation for Statistical Computing, Vienna, Austria).

#### 2.3.2 Variable description and screening

Continuous variables were analyzed using independent sample *t*-tests and are presented as mean ± standard deviation (SD); Categorical variables were analyzed using the chi-square (χ^2^) test and are reported as frequency and percentage [*n* (%)]. Univariate analysis was conducted for each candidate variable to assess its association with ENFI. Variables with *p* < 0.05 in univariate analysis were further assessed for multicollinearity using the variance inflation factor (VIF). A VIF > 5 was considered indicative of significant multicollinearity and such variables were excluded from multivariate analysis.

Dichotomous variables were coded as 0 or 1 for entry into the regression model, while continuous variables were included in their original numeric form. Variables that met the inclusion criteria were entered into a binary logistic regression model. Forward stepwise regression was employed to identify independent predictors of ENFI in ICU patients.

#### 2.3.3 Nomogram and evaluation

Based on the results of the binary logistic regression analysis, a visual nomogram was constructed to predict the risk of enteral nutrition feeding intolerance (ENFI) in ICU patients. The model’s discriminative ability and predictive accuracy were subsequently evaluated. Internal validation was conducted using the bootstrap resampling method, which involved drawing 1,000 resamples from the original dataset. The development of a robust clinical prediction model typically involves three key stages: model construction, internal validation, and external validation. Internal validation assesses model performance using data that are similar to the original dataset used for model development, whereas external validation evaluates the model using data from different populations or settings (e.g., multi-center datasets), thereby testing its generalizability. In this study, all data were obtained from ICU patients in the Jining region. As the internal validation population was derived from the same source as the model development population, the dataset was not split into training and validation subsets. Instead, the bootstrap method was employed to perform internal validation and assess the stability and calibration of the predictive model.

The model’s ability to distinguish between patients with and without ENFI was evaluated using the area under the receiver operating characteristic curve (AUC). Additionally, the sensitivity, specificity, positive predictive value (PPV), and negative predictive value (NPV) of the model were calculated.

For calibration, a calibration curve was plotted comparing predicted probabilities against observed outcomes. The Hosmer–Lemeshow goodness-of-fit test was conducted to assess the agreement between predicted and actual outcomes. A *p*-value > 0.05 indicated good calibration and acceptable predictive performance.

## 3 Results

### 3.1 Basic information about the research subjects

A total of 144 ICU patients were enrolled in this study, including 87 patients with feeding tolerance (FT) and 57 patients who developed ENFI, yielding an overall ENFI incidence of 39.6%. Among patients in the ENFI group, males were more prevalent, the majority had chronic comorbidities, and nearly two-thirds had undergone mechanical ventilation and presented with serum albumin levels below 35 g/L. A detailed comparison of baseline demographic and clinical characteristics between the ENFI and non-ENFI groups is provided in Table 1.

**Table 1:** Baseline characteristics of ICU patients in the FT and FI groups.

Items	FT group (*n* = 87)	FI group (*n* = 57)	t/χ^2^	*P*-value
Age	66.31 ± 17.53	65.21 ± 14.77	0.391	0.295
Gender			0.240	0.624
Male	52 (59.8%)	36 (63.2%)		
Female	35 (40.2%)	21 (36.8%)		
BMI			1.308	0.253
<24	48 (55.2%)	36 (63.2%)		
≥24	39 (44.8%)	21 (36.8%)		
APACHE II score			18.936	<0.001
≤20	59 (67.8%)	21 (36.8%)		
>20	28 (32.2%)	36 (63.2%)		
Combined chronic diseases			0.006	0.940
Yes	63 (72.4%)	41 (71.9%)		
No	24 (27.6)	16 (28.1%)		
MV			19.990	<0.001
Yes	37 (42.5%)	42 (73.7%)		
No	50 (57.5%)	15 (26.3%)		
Antibiotics			1.459	0.227
Yes	54 (62.1%)	40 (70.2%)		
No	33 (37.9%)	17 (29.8%)		
Sedative analgesics			0.190	0.663
Yes	50 (57.5%)	31 (54.4%)		
No	37 (42.5%)	26 (45.6%)		
Potassium preparations			1.869	1.172
Yes	58 (66.7%)	43 (75.4%)		
No	29 (33.3%)	14 (24.6%)		
ALB			17.303	<0.001
≤35 g/l	39 (44.8%)	42 (73.7%)		
>35 g/l	48 (55.2%)	15 (26.3%)		
NRS2002 score	2.91 ± 1.11	3.05 ± 1.06	0.372	0.543
Aspiration risk score	23.25 ± 4.42	24.32 ± 3.07	5.573	0.016
IAP			17.149	<0.001
<12 mmHg	69 (79.3%)	29 (50.9%)		
≥12 mmHg	18 (20.7%)	28 (49.1%)		
EN start time			9.264	0.002
≤48 h	41 (47.1%)	39 (68.4%)		
>48 h	46 (52.6%)	18 (31.6%)		
EN feeding methods			0.249	0.617
Nasogastric tube	85 (97.7%)	55 (96.5%)		
Nasojejunal tube	2 (2.3%)	2 (3.5%)		

APACHE II score, Acute Physiology and Chronic Health Evaluation II score; MV, mechanical ventilation; ALB, albumin; IAP, intra-abdominal pressure; EN, enteral nutrition.

### 3.2 Screening variables

#### 3.2.1 Univariate analysis

The χ^2^ test and independent sample *t*-test were used to compare potential predictor variables between the ENFI and non-ENFI groups. The results indicated that the APACHE II score, MV, ALB, aspiration risk score, IAP, and EN start time were all significantly associated with ENFI (*p* < 0.05). Detailed results are presented in Table 1.

#### 3.2.2 Multicollinearity test and variable assignment

Before conducting binary logistic regression analysis, multicollinearity diagnostics were performed on the six variables found to be statistically significant in univariate analysis. Categorical variables were appropriately coded, and continuous variables were retained in their original form. The results showed that all VIF values were less than 5, indicating no significant multicollinearity among the variables. Therefore, all six predictors—APACHE II score, MV, ALB, aspiration risk score, IAP, and EN start time—were included in the binary logistic regression model. Detailed multicollinearity results are presented in Table 2.

**Table 2:** Multicollinearity diagnostics and variable assignment for logistic regression.

Risk factors	Tolerance	VIF	Assignment
APACHE II score	0.822	1.217	0 ≤ 20; 1>20
MV	0.860	1.163	0 = “No”; 1 = “Yes”
ALB	0.913	1.095	0>35; 1 ≤ 35
Aspiration risk score	0.908	1.101	Original value entry
IAP	0.929	1.077	0 < 12; 1 ≥ 12;
EN start time	0.989	1.011	0 ≤ 48; 1 > 48;

APACHE II score, Acute Physiology and Chronic Health Evaluation II score; MV, mechanical ventilation; ALB, albumin; IAP, intra-abdominal pressure; EN, enteral nutrition.

#### 3.2.3 Binary logistic regression analysis

A binary logistic regression analysis was conducted using ENFI status as the dependent variable. The six statistically significant variables identified in the univariate analysis were included as independent variables. The results showed that APACHE II score, MV, ALB, IAP, and EN start time were independently associated with the occurrence of ENFI (*p* < 0.05). These findings indicate that these five factors are independent predictors of ENFI in ICU patients. Detailed regression results are presented in Table 3.

**Table 3:** Results of binary logistic regression analysis.

Risk factors	B	SE	Wals	*P*	OR	95% CI
APACHE II score	0.755	0.348	4.713	0.03	2.128	1.076–4.810
MV	1.062	0.351	9.178	0.002	2.892	1.455–5.750
ALB	0.896	0.344	6.781	0.009	2.450	1.248–4.810
Risk of aspiration score	0.000	0.046	0.000	0.992	1.000	0.914–1.095
IAP	1.178	0.344	6.781	0.009	2.450	1.248–4.810
EN start time	−0.993	0.337	8.684	0.003	0.371	0.191–0.717

APACHE II score, Acute Physiology and Chronic Health Evaluation II score; MV, mechanical ventilation; ALB, albumin; IAP, intra-abdominal pressure; EN, enteral nutrition.

### 3.3 Construct and evaluate the nomogram

#### 3.3.1 Establishing the nomogram

**Figure 1:**
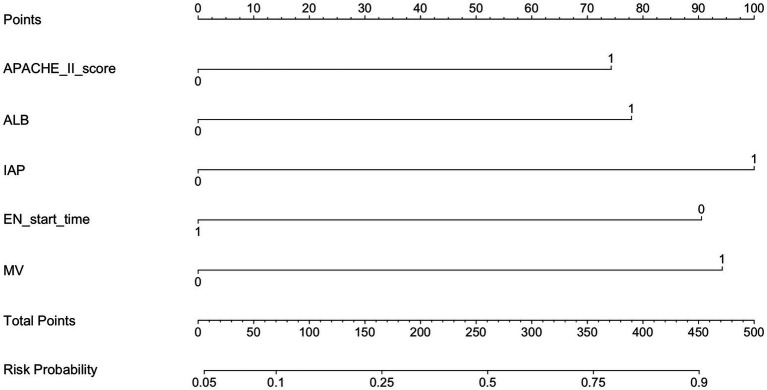
Predicting the risk of ENFI in the nomogram. APACHE II score, Acute Physiology and Chronic Health Evaluation II score; MV, mechanical ventilation; ALB, albumin; IAP, intra-abdominal pressure; EN, enteral nutrition.

Based on the results of the binary logistic regression analysis, a nomogram was constructed to visually represent the predictive model, as shown in Figure 1. The nomogram incorporates five independent predictors—APACHE II score, MV, ALB, IAP, and EN Start time. Each variable is assigned a corresponding point value, and the total score is calculated by summing the points for all five indicators. The total score corresponds to the estimated probability of ENFI in ICU patients.

#### 3.3.2 Evaluation of the nomogram

**Figure 2:**
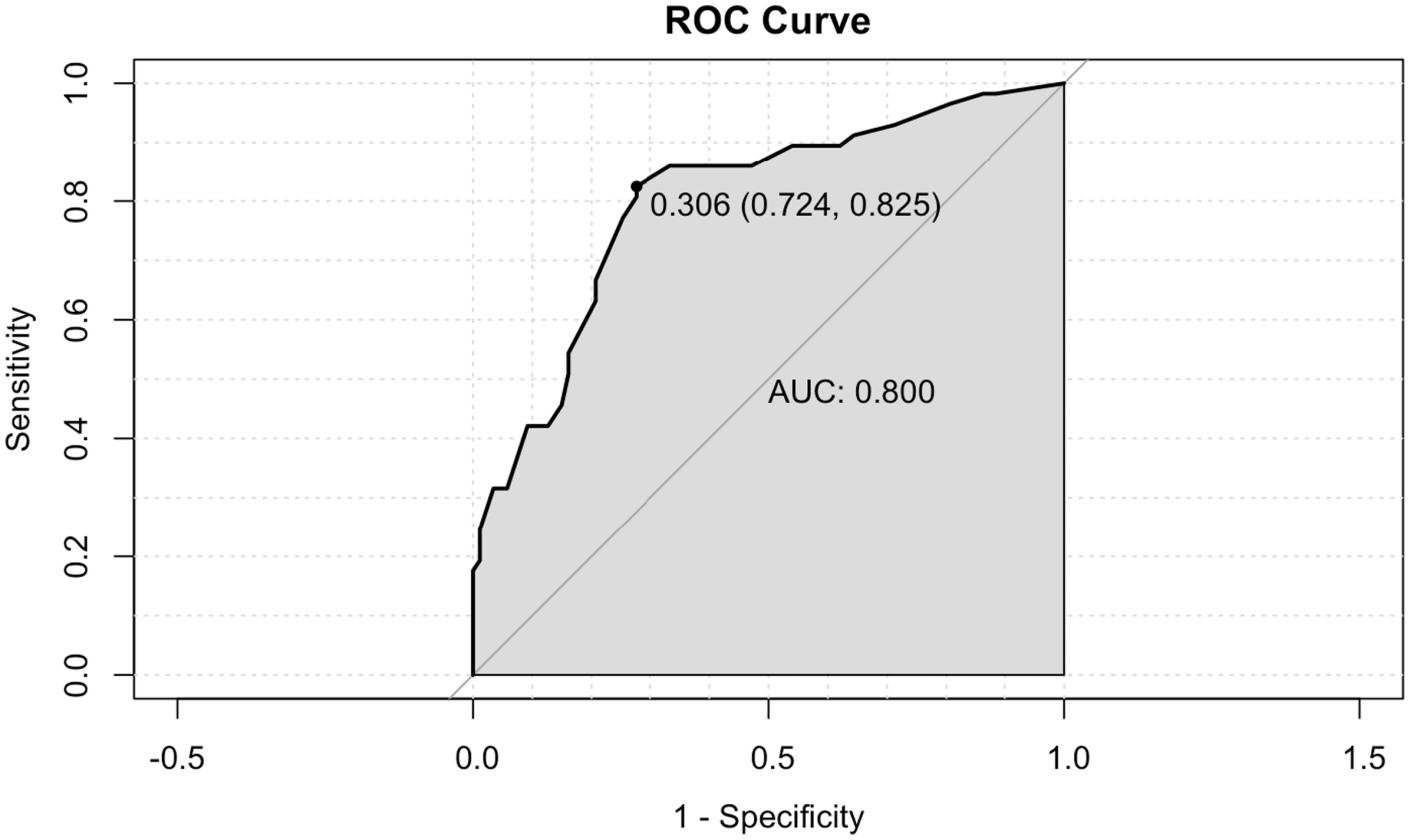
ROC curves for the risk prediction model of ENFI.

The discriminative ability of the nomogram model was evaluated using the AUC, as illustrated in Figure 2. The AUC was 0.800 (95% confidence interval: 0.725–0.875), with an optimal cutoff value of 0.306. At this threshold, the model demonstrated a sensitivity of 82.5%, a specificity of 72.4%, a PPV of 67.2%, and a NPV of 86.3%. These results indicate that the nomogram model has good discriminative performance in identifying ICU patients at risk of ENFI.

**Figure 3:**
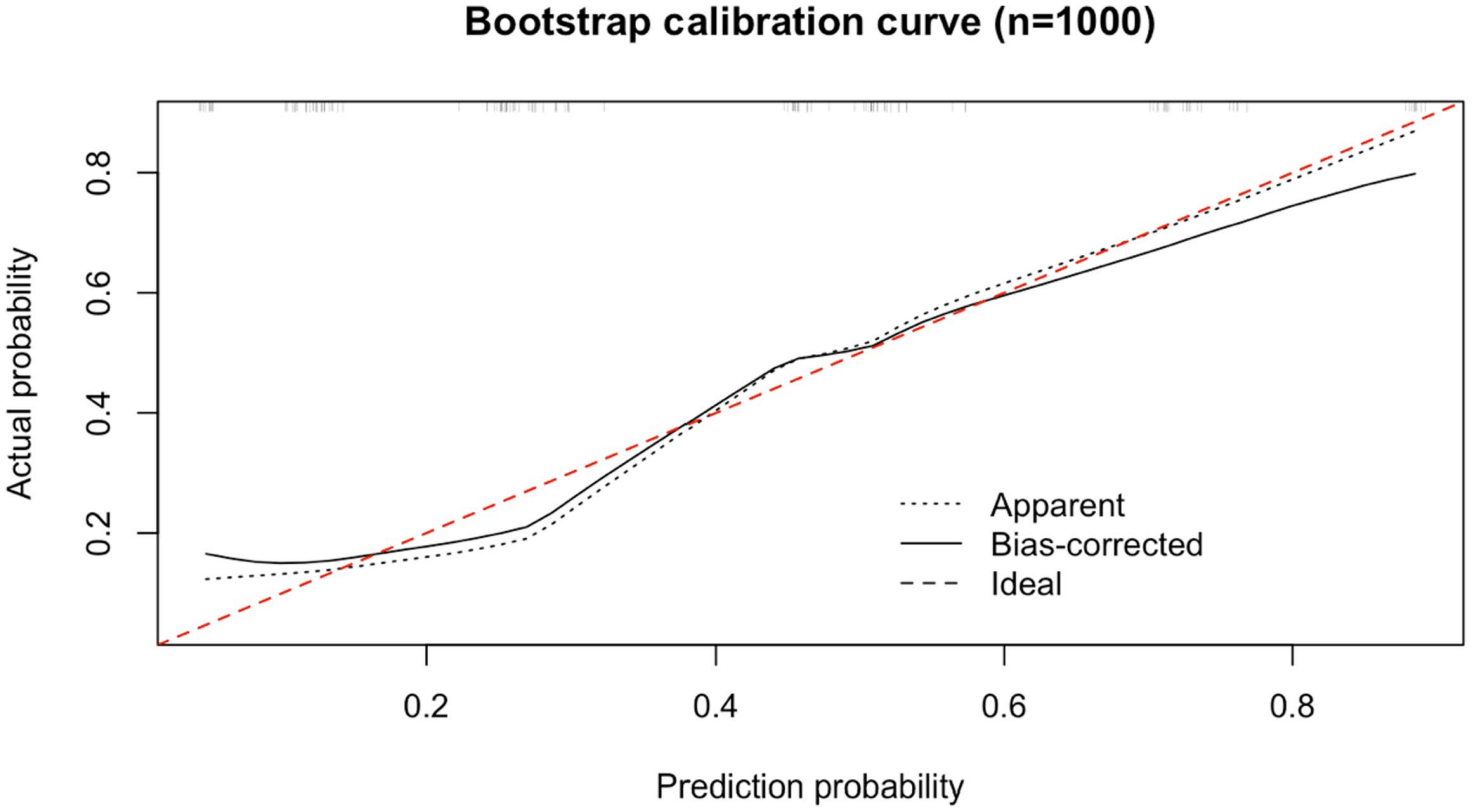
Calibration curves for the nomogram model predicting the risk of developing ENFI.

Internal validation of the nomogram was performed using the bootstrap method. Following 1,000 resamplings of the original dataset, a calibration curve was generated, as shown in Figure 3. The Hosmer–Lemeshow goodness-of-fit test yielded a χ^2^ value of 2.9954 with a *P* of 0.9346, indicating no statistically significant difference between the predicted probabilities and the actual observed outcomes. This result suggests that the model demonstrates strong agreement between predicted and actual risk of ENFI, reflecting good calibration and predictive accuracy.

## 4 Discussion

This study identified five independent risk factors for enteral nutrition feeding intolerance (ENFI) in ICU patients using binary logistic regression analysis: APACHE II score, MV, ALB, IAP, and EN start time. These factors demonstrated significant associations with the occurrence of ENFI, suggesting that systematic assessment of these parameters may facilitate early identification of high-risk patients and enable timely intervention to improve nutritional tolerance and clinical outcomes. The specific findings are discussed in detail below:

### 4.1 APACHE II score

The results of this study indicate that critically ill patients with an APACHE II score > 20 are significantly more likely to develop enteral nutrition feeding intolerance (ENFI), with an odds ratio of 2.128 compared to those with a score ≤ 20. This finding is consistent with previous research by Wang et al. (16), who also identified a high APACHE II score as an independent predictor of ENFI. Additionally, our study shows that the risk of ENFI in patients with an APACHE II score > 20 is 2.128 times higher than in those with an APACHE II score ≤ 20.

The APACHE II score is a widely used clinical tool to assess the severity of illness in ICU patients; higher scores are associated with greater physiological derangement, increased stress response, and poorer outcomes (17). Elevated APACHE II scores reflect a heightened systemic inflammatory and metabolic response, which may negatively impact gastrointestinal function. Under such stress conditions, patients often exhibit reduced gastrointestinal perfusion, impaired peristalsis, and compromised mucosal barrier integrity. These pathophysiological changes lead to delayed gastric emptying, increased intestinal permeability, and ultimately reduced tolerance to enteral nutrition (15, 18). Several studies support the relationship between high APACHE II scores and ENFI risk. For example, Bolayir et al. (18) found that an APACHE II score greater than 20 was significantly associated with gastrointestinal intolerance in critically ill stroke patients.

Given this evidence, ICU clinicians, particularly nurses and dietitians, should pay close attention to such patients. In addition to actively treating the underlying disease and stabilizing the condition, prokinetic agents can be used to prevent the occurrence of FI, ensuring the smooth implementation of enteral nutrition.

### 4.2 ALB

In this study, the majority of patients in the ENFI group had hypoalbuminemia (ALB ≤ 35 g/L), and multivariate analysis confirmed that low albumin was an independent risk factor for ENFI, with an odds ratio (OR) of 2.450 (*p* = 0.009). Albumin is a key biomarker reflecting both nutritional status and systemic inflammation. Adequate albumin levels contribute to enhanced gastrointestinal tolerance through several physiological mechanisms. McClave et al. (4) reported that ICU patients with albumin levels ≥30 g/L exhibited significantly lower gastric residual volumes and achieved target enteral feeding rates more rapidly compared to those with lower albumin levels. Albumin helps maintain plasma colloid osmotic pressure, which can effectively reduce intestinal wall edema and support the recovery of gastrointestinal mucosal integrity (19, 20). Moreover, albumin has anti-inflammatory and detoxifying functions—it can bind and neutralize circulating endotoxins, thereby mitigating intestinal inflammation and reducing the risk of feeding-related complications (21). These protective mechanisms highlight the multifaceted role of albumin in supporting enteral nutrition tolerance.

Given these findings, close monitoring of serum albumin levels is essential in ICU patients receiving EN. For those with ALB ≤ 35 g/L, timely nutritional interventions, including protein supplementation and inflammation management, may improve EN tolerance and reduce the incidence of ENFI.

### 4.3 IAP

In this study, nearly half of the patients in the ENFI group exhibited IAP ≥ 12 mmHg, identifying elevated IAP as an independent risk factor for ENFI. IAP refers to the pressure within the abdominal cavity, and the gastrointestinal tract is among the most sensitive organs to elevated IAP (22). Increased IAP can reduce mesenteric perfusion, compress the thin-walled mesenteric veins, and obstruct intestinal venous return, which leads to intestinal edema, impaired mucosal barrier function, and delayed gastrointestinal motility and emptying (23).

Moreover, a bidirectional relationship may exist: while elevated IAP can impair gastrointestinal function, compromised intestinal digestion and delayed gastric emptying can in turn exacerbate IAP, forming a vicious cycle (24, 25). Thus, IAP functions as both a cause and consequence of ENFI, forming a self-perpetuating pathophysiological loop. Bejarano et al. (26) demonstrated that IAP is negatively correlated with enteral nutrition tolerance, and elevated IAP values can be used as a predictive marker for ENFI in critically ill patients.

Measurement of IAP via bladder pressure is widely recognized as the gold standard due to its simplicity, non-invasiveness, reliability, and minimal interference from external variables (27). In this study, IAP was regularly monitored using bladder pressure measurements as part of routine ICU care. When elevated IAP was detected, clinical staff proactively investigated potential causes—such as constipation, abdominal distension, fluid overload, or mechanical ventilation settings—and implemented timely interventions to mitigate further gastrointestinal compromise.

### 4.4 EN start time

The results of this study indicate that the timing of EN initiation is an independent risk factor for ENFI, with a later start of EN associated with a reduced risk of developing feeding intolerance. This finding challenges the traditional emphasis on initiating EN as early as possible in all critically ill patients (3, 15, 28).

According to the 2019 ESPEN clinical nutrition guidelines for intensive care units, early initiation of EN—typically within 24 to 48 h—is recommended primarily to preserve gut integrity and reduce infection complications in patients without contraindications (3). However, the same guidelines also emphasize that full-dose EN should not be started too early in critically ill patients to avoid the risk of overfeeding and associated complications. The guidelines suggest that a gradual advancement of caloric and protein targets over the first 3 to 7 days may be safer and more physiologically appropriate. Furthermore, Singer et al. (3) reported that while early EN may reduce infection-related complications in non-ICU patients, this benefit is not as clearly observed in ICU populations. As such, overly aggressive early EN in hemodynamically unstable or severely ill ICU patients may increase the risk of ENFI due to impaired gastrointestinal tolerance. In this context, delaying the initiation of EN beyond 48 h, until the patient’s condition stabilizes, may improve tolerance and reduce gastrointestinal complications.

These findings highlight the need for individualized EN strategies based on patient condition, rather than strict adherence to early EN protocols. Clinical staff should evaluate factors such as gastrointestinal function, hemodynamic status, and nutritional risk when determining the optimal time to initiate EN.

### 4.5 MV

In this study, the incidence of ENFI among patients receiving MV was 73.7%, and MV was identified as an independent risk factor for ENFI. Patients who underwent MV were 2.892 times more likely to develop ENFI compared to those who did not, consistent with the findings of Li et al. (29). This highlights a strong association between MV and the development of feeding intolerance in critically ill patients.

MV is a common supportive therapy in the ICU, but it may adversely affect gastrointestinal function through multiple mechanisms. Positive-pressure ventilation can cause air to enter the digestive tract, resulting in gastric distension and impaired gastrointestinal motility (22). Furthermore, factors such as positive end-expiratory pressure (PEEP) can compromise splanchnic perfusion, leading to reduced gastrointestinal blood flow and elevated IAP (30). As noted previously, elevated IAP is itself an independent risk factor for ENFI.

In addition, patients receiving MV frequently require sedatives, analgesics, and vasoactive agents. Sedative and analgesic drugs, such as benzodiazepines and opioids, are known to suppress gastrointestinal motility, thereby increasing the risk of delayed gastric emptying and FI (31). Meanwhile, vasoactive medications may induce gastrointestinal ischemia and hypoperfusion, further exacerbating mucosal damage and dysfunction.

In conclusion, MV not only independently contributes to the risk of ENFI but also acts through multiple synergistic pathways that impair gastrointestinal function and tolerance to enteral feeding. In clinical practice, special attention should be paid to patients undergoing MV. It is essential to maintain appropriate endotracheal cuff pressures, monitor gastrointestinal symptoms closely, assess abdominal and respiratory parameters dynamically, and aim to wean patients from ventilatory support as early as clinically feasible to reduce the risk of ENFI.

### 4.6 Analysis of other variables

In this study, no statistically significant differences were observed between the ENFI and non-ENFI groups in terms of the following variables: age, gender, BMI, combined chronic diseases, antibiotics, NRS 2002, sedative analgesics, potassium preparations, and EN feeding methods. The lack of significant differences may indicate that the development of ENFI is more strongly influenced by acute pathophysiological factors (e.g., disease severity, intra-abdominal pressure, mechanical ventilation status) rather than baseline demographic or general clinical characteristics. However, further multicenter studies with larger sample sizes are warranted to confirm these findings and explore potential interactions. The specific analysis is as follows:

Aging is known to be associated with a decline in gastrointestinal function, including slower gastric emptying and reduced motility. Horowitz et al. (32) demonstrated that advancing age is correlated with delayed gastric emptying. Additionally, gender-based physiological differences have been observed, with studies showing that healthy women have slower gastric motility compared to men (33).

However, in the present study, no statistically significant differences in age or gender were observed between the ENFI and non-ENFI groups. One possible explanation is that all study participants were critically ill, and the acute severity of illness may have outweighed the influence of baseline demographic factors. In such patients, pathophysiological disturbances such as systemic inflammation, organ failure, and hemodynamic instability may play a more dominant role in gastrointestinal dysfunction than age-related decline.

While several previous studies have identified advanced age as an independent risk factor for ENFI (7, 34, 35), other studies have failed to find a significant association (36, 37). These inconsistencies suggest that the relationship between age and ENFI may be context-dependent and influenced by factors such as study population, illness severity, and comorbidities. Therefore, further research is warranted to clarify the role of age as an independent predictive factor, particularly in heterogeneous populations of critically ill patients in the ICU.

Antibiotics not only eradicate pathogenic bacteria but also disrupt the balance of beneficial gut microbiota, leading to gastrointestinal dysbiosis and contributing to the development of FI (38). In this study, the use of potassium supplements was also recorded. Potassium preparations are known to directly irritate the gastrointestinal mucosa and may induce symptoms such as nausea, vomiting, abdominal pain, and diarrhea, thereby diminishing patients’ appetite and tolerance to enteral nutrition (39). Although a large proportion of patients in this study had chronic underlying diseases, the specific types of comorbidities were not stratified or analyzed in detail, which represents a limitation. Previous research has shown that the incidence of FI can vary significantly across different disease populations. Research (40) reported that patients with burns, gastrointestinal disorders, sepsis, and cardiovascular diseases exhibit higher rates of FI compared to those with respiratory diseases. These findings underscore the importance of considering disease-specific factors in FI risk assessments and highlight the need for future studies to include more detailed clinical classifications of comorbid conditions.

In this study, the NRS 2002 score was not found to be a statistically significant factor associated with FI. However, a large-scale, multicenter, longitudinal database study conducted by Heyland et al. (41) reported significant differences in severe nutritional risk scores between patients with and without FI, suggesting a potential association. Despite its widespread use in nutritional screening, there is currently limited research specifically examining the predictive value of the NRS 2002 score for FI. This tool was primarily developed to assess overall nutritional risk rather than gastrointestinal tolerance. Therefore, its utility in predicting FI in critically ill patients remains uncertain. Further prospective studies are needed to determine whether the NRS 2002 score can reliably identify patients at higher risk of developing FI and whether it should be integrated into FI risk prediction models.

In clinical practice, nasogastric and nasojejunal tubes are commonly used for short-term enteral nutrition (EN) in patients requiring nutritional treatment for less than 6 weeks. A study (42) showed that nasojejunal feeding is associated with a lower incidence of gastric residuals, reduced risk of reflux, aspiration, and diarrhea, and improved nutrient absorption, compared to nasogastric feeding. These findings suggest that the choice of feeding route may influence the risk of enteral nutrition feeding intolerance (ENFI). In the present study, nasogastric tubes were the predominant feeding route, which may have influenced the observed outcomes. However, no statistically significant difference in ENFI incidence was found between different feeding routes (*p* > 0.05). This may be attributed to the relatively small sample size or limited use of nasojejunal tubes, which may have reduced the power to detect a difference. Future studies with a more balanced distribution of feeding routes and larger sample sizes are needed to further explore the impact of tube placement on feeding intolerance in critically ill patients.

### 4.7 Predictive performance of the model

In this study, the incidence of enteral nutrition feeding intolerance (ENFI) was 39.6%, which is consistent with the rates reported in clinical guidelines (43). Generally, the area under the ROC curve (AUC) is used to evaluate the diagnostic or predictive performance of a model: an AUC between 0.5 and 0.7 indicates low accuracy, 0.7 to 0.9 suggests moderate accuracy, and an AUC greater than 0.9 reflects high accuracy. The AUC of the predictive model developed in this study was 0.800, indicating moderate discriminatory ability and suggesting that the model has practical clinical value. Based on the optimal Youden index, a cutoff probability of 30.6% was identified to classify a positive prediction of ENFI, which balanced the sensitivity (82.5%) and specificity (72.4%) of the model. Overall, the risk prediction model constructed for ICU patients demonstrates good predictive performance and can effectively assist clinicians in early identification and intervention for patients at high risk of developing ENFI.

This study offers a simple and visual prediction model in the form of a nomogram that enables early risk stratification for ENFI in ICU patients. By assessing five routinely available clinical variables, clinicians can estimate the likelihood of feeding intolerance and adjust nutritional strategies accordingly. For high-risk patients, measures such as slower feeding advancement, alternative feeding routes, prophylactic prokinetic agents, or closer gastrointestinal monitoring may be considered to enhance EN tolerance and avoid complications. The model may also support communication with multidisciplinary teams and facilitate decision-making during ICU rounds.

## 5 Limitations

The study excluded patients with gastrointestinal diseases and those who had undergone abdominal surgery, which may introduce some bias when using this model to predict outcomes for such patients. Although the sample size falls within the theoretically calculated range, the number of included patients remains relatively small, which may affect the model’s accuracy. Additionally, since the data used for internal validation is the same as that in this study, no external validation was conducted. Furthermore, subgroup analyses or interaction effect assessments (e.g., by age, sex, or disease category) were not performed. These analyses would be valuable in determining whether the model performs consistently across different clinical subpopulations. Moreover, chronic comorbidities were treated as a binary variable (yes/no), without distinguishing between specific disease types such as cardiovascular disease, diabetes, or renal failure. This simplified classification may obscure important sources of heterogeneity in ENFI risk and reduce the model’s explanatory power. Future studies should aim for a more detailed classification of comorbid conditions, along with expanded sample sizes through multicenter research, incorporate subgroup analysis, and conduct external validation to improve the model’s precision and generalizability.

## 6 Conclusion

This study developed a predictive model for enteral nutrition feeding intolerance (ENFI) in ICU patients, incorporating five routinely assessed clinical variables: APACHE II score, MV, ALB, IAP, and EN start time. The model demonstrated favorable predictive performance, with good discrimination and calibration. Given the accessibility and clinical relevance of these variables, the model may be preliminarily applied for early risk stratification of ENFI in ICU settings. Its simplicity enables healthcare providers to identify high-risk patients early, potentially informing preventive measures and individualized nutritional strategies. These findings underscore the multifactorial etiology of ENFI and the importance of a multidisciplinary approach involving physicians, dietitians, and nursing staff.

However, as this was an observational study, causal relationships cannot be established, and the model’s findings should be interpreted with caution. Further external validation in larger, multicenter cohorts is warranted to confirm its generalizability and utility. With such validation, the model could serve as a useful reference to support nutritional management and improve patient outcomes in critically ill populations.

## Data Availability

The raw data supporting the conclusions of this article will be made available by the authors, without undue reservation.
